# Animal board invited review: genetic possibilities to reduce enteric methane emissions from ruminants

**DOI:** 10.1017/S1751731115000968

**Published:** 2015-06-09

**Authors:** N. K. Pickering, V. H. Oddy, J. Basarab, K. Cammack, B. Hayes, R. S. Hegarty, J. Lassen, J. C. McEwan, S. Miller, C. S. Pinares-Patiño, Y. de Haas

**Affiliations:** 1 Animal Productivity, AgResearch, Invermay Agricultural Centre, Puddle Alley, PB50034, Mosgiel 9010, New Zealand; 2 NSW Department of Primary Industries, Beef Industry Centre, University of New England, Armidale NSW 2351, Australia; 3 Alberta Agriculture and Rural Development, Lacombe Research Centre, 6000 C & E Trail, Lacombe, AB, Canada T4L 1W1; 4 Department of Animal Science, University of Wyoming, Laramie, Wyoming 82071, USA; 5 Biosciences Research Division, Department of Environment and Primary Industries, Bundoora 3083, Victoria, Australia; 6 Dairy Futures Cooperative Research Centre, Bundoora 3083, Victoria, Australia; 7 La Trobe University, Bundoora, Victoria, Australia; 8 University of New England, Armidale NSW, Australia; 9 Center for Quantitative Genetics and Genomics, Institute of Molecular Biology and Genetics, Aarhus University, Denmark; 10 Centre for the Genetic Improvement of Livestock, University of Guelph, Guelph, Ontario, Canada; 11 Livestock Gentec, University of Alberta, Edmonton, Alberta, Canada; 12 Animal Nutrition & Health, AgResearch, Grasslands Research Centre, Tennent Drive, PB 11008, Palmerston North, New Zealand; 13 Animal Breeding and Genomics Centre of Wageningen UR Livestock Research, P.O. Box 135, 6700 AC Wageningen, the Netherlands

**Keywords:** genetics, greenhouse gases, enteric methane, ruminants

## Abstract

Measuring and mitigating methane (CH_4_) emissions from livestock is of increasing importance for the environment and for policy making. Potentially, the most sustainable way of reducing enteric CH_4_ emission from ruminants is through the estimation of genomic breeding values to facilitate genetic selection. There is potential for adopting genetic selection and in the future genomic selection, for reduced CH_4_ emissions from ruminants. From this review it has been observed that both CH_4_ emissions and production (g/day) are a heritable and repeatable trait. CH_4_ emissions are strongly related to feed intake both in the short term (minutes to several hours) and over the medium term (days). When measured over the medium term, CH_4_ yield (MY, g CH_4_/kg dry matter intake) is a heritable and repeatable trait albeit with less genetic variation than for CH_4_ emissions. CH_4_ emissions of individual animals are moderately repeatable across diets, and across feeding levels, when measured in respiration chambers. Repeatability is lower when short term measurements are used, possibly due to variation in time and amount of feed ingested prior to the measurement. However, while repeated measurements add value; it is preferable the measures be separated by at least 3 to 14 days. This temporal separation of measurements needs to be investigated further. Given the above issue can be resolved, short term (over minutes to hours) measurements of CH_4_ emissions show promise, especially on systems where animals are fed *ad libitum* and frequency of meals is high. However, we believe that for short-term measurements to be useful for genetic evaluation, a number (between 3 and 20) of measurements will be required over an extended period of time (weeks to months). There are opportunities for using short-term measurements in standardised feeding situations such as breath ‘sniffers’ attached to milking parlours or total mixed ration feeding bins, to measure CH_4_. Genomic selection has the potential to reduce both CH_4_ emissions and MY, but measurements on thousands of individuals will be required. This includes the need for combined resources across countries in an international effort, emphasising the need to acknowledge the impact of animal and production systems on measurement of the CH_4_ trait during design of experiments.

## Implication

Measuring and mitigating methane (CH_4_) emissions from livestock is of increasing importance for the environment and for policy making. Potentially, the most sustainable way of reducing enteric CH_4_ emission from ruminants is through the estimation of genomic breeding values to facilitate genetic selection. Enteric CH_4_ emissions are difficult and expensive to measure, thus genomic prediction could provide significant, long-term economic benefits. Implementation will require global collaboration to define a suitable measure and many thousands of records to ensure valid and accurate evaluations.

## Introduction

Climate change is of growing international concern and it is well established that the release of greenhouse gases (GHG) is the driving factor (IPCC, 2006). Globally, livestock farming contributes ~9% to 11% of total anthropogenic GHG emissions (Smith *et al.*, 2007; Tubiello *et al.*, 2013). Of the various GHG, methane (CH_4_) is the most important agricultural contributor, with a global warming potential 25 times that of carbon dioxide (CO_2_) (Forster *et al.*, 2007).

Globally, in the year 2010, GHG emissions from the agriculture sector accounted for 4.6 GtCO_2_ eq, of which enteric fermentation (emissions of CH_4_ from ruminant animals) contributed 2 GtCO_2_ eq (Tubiello *et al.*, 2013), with an annual increase of 0.95% between 1961 and 2010. Non-dairy cattle were the single largest source of enteric CH_4_, followed by dairy cattle, buffaloes, sheep and goats (FAOSTAT, 2013). Enteric CH_4_ emissions from ruminant livestock (cattle, sheep and goats) account for 2% to 12% of gross energy intake (Blaxter, 1962; Johnson and Johnson, 1995). Although CH_4_ production is an energy loss to ruminants, it can also be considered a small price to pay for their adaptation to digest cellulose-based feeds. Sources of systematic variation in CH_4_ production by an individual animal include: total feed intake, the nutrient composition of the feed eaten, the proportion and rate of fermentation of that feed in the rumen, feeding frequency (for recent reviews see Hristov *et al.*, 2013a and 2013b), rumen volume and rate of passage of digesta from the rumen (Goopy *et al.*, 2014), physiological state of the animal and variation between individual animals including that between sire families (Pinares-Patiño *et al.*, 2013a).

Production of CH_4_ (and other GHGs) per unit of animal product (e.g. milk, meat) has declined over the past 50 years in most ruminant livestock industries in developed countries due to ongoing improvements in animal productivity. For example, the carbon footprint, in terms of CO_2_ eq/kg of milk produced, of the US dairy industry in 2007 was 37% of that in 1944 (Capper *et al.*, 2009). Productivity improvements included a change of breed type of the dairy cow (to Holstein), improved genetics within the Holstein breed and a shift from a forage based to total mixed ration feeding system (see Capper *et al.*, 2009). Similarly, analysis of the carbon footprint of total US beef production indicates a reduction of CO_2_ eq of 16% per kg of beef produced in 2007 compared with 1977 (Capper, 2011), due to a reduction in total feedstuff used, changed industry structure, improved nutritional management and improved herd genetics.

Most of the mitigation potential in the livestock sector is found in the developing countries. However, for these countries, it is important to combine development and mitigation strategies, like adapted selection programmes and feeding strategies, as a lot can still be achieved in developing countries by increasing lifetime production of animals (Gerber *et al.*, 2013).

The extent to which genetic improvement can contribute to improvement in individual animal milk production and consequent impacts on GHG emissions has been highlighted by Wall *et al.* (2010). They described how systematic improvement in environmental outcomes has resulted from productivity improvements and discussed how direct and indirect measures of emissions can be incorporated into breeding objectives to reduce emissions.

There are many potential methods to reduce enteric CH_4_ emissions per head and thereby intensity of CH_4_ production per unit product. These include: changing feed type (e.g. from pasture to concentrate feed or to new pasture varieties); use of supplements that reduce CH_4_ emissions (fats, oils, plant extracts and nitrate); improving productivity through management change including use of growth enhancers and improved genetics; immunisation against methanogens and selective breeding of animals with low CH_4_ emissions, through either reduced feed intake per product or reduced CH_4_ production per feed consumed, without compromising production characteristics (Martin *et al.*, 2010; Wall *et al.*, 2010). The aim of this review is to provide an overview of possibilities and some of the remaining issues that need to be addressed to realise these possibilities to genetically reduce enteric CH_4_ emissions by livestock.

## Quantifying enteric CH_4_ emission

There are three levels in which a CH_4_ trait can be defined; first, the farm system level which uses information on the number of animals present within a system boundary with a related estimate of CH_4_ emissions per head, calculated for example from the Intergovernmental Panel on Climate Change (2006) Tier 2 calculations. These calculations have embedded within them a number of assumptions about the factors which affect CH_4_ emission per head, that is feed intake, feed quality and CH_4_ yield. Second, the animal production level which uses information about productivity per head that is milk yield or kg carcass weight, from individual animals to give us CH_4_ intensity (g CH_4_/kg product). Finally, at the animal level, individual CH_4_ emissions and feed intake measurements to enable genetic progress on CH_4_ yield (MY; g CH_4_/kg dry matter intake (DMI)), or residual feed intake (RFI; MJ/day), which is the difference between net energy intake and calculated energy requirements for maintenance as a function of live weight and for fat and protein corrected milk yield.

## Methodologies for measurement of CH_4_ from ruminants

The respiratory chamber (RC) system is often viewed as a ‘gold standard’ for emission measurement. There is little question RC measurements accurately quantify CH_4_ output over the 1 to 3-day measurement period typically used, and they achieve this by frequently measuring emissions. The variability in emission rate resulting from eructation events, animal position and feed intake that occur in 24 h, are typically damped within the large chamber volume. Feeding in RCs can also cause a reduction in feed intake (relative to pre-chamber intakes) and completely eliminates diet selection and feeding pattern which has strong genetic control and may well be a means by which animal genetics moderates emission in the grazing environment (Hegarty, 2004). The RCs rarely monitor CH_4_ outflow on a second by second basis, the chambers used to estimate CH_4_ parameters do so by measuring volume of air flow coupled with intermittent sampling (at 3 to 13 min) of gas for determination of CH_4_ concentrations. This means that hourly measurements described here consist of averages of 4 to 20 measurements each taken over a few seconds (albeit averaged via dilution in a large volume that is the chamber). As shown by Pinares-Patiño *et al.* (2013a) a 1 to 3-day collection only poorly describes the CH_4_ phenotype of an animal over a year or a lifetime and could benefit from repeated measurements. In reality, CH_4_ is largely emitted intermittently via brief eructations or burps lasting only seconds, albeit with a basal level of emission.

The sulfur hexafluoride (SF_6_) technique is one tool that offers field measurement over a longer time, but requires insertion of rumen boluses, daily animal handling and laboratory measurement of gases (McGinn *et al.*, 2006). Moreover, the sampling procedures provide an average CH_4_ output for periods of typically 24 h, but can be repeated over periods of 5 to 10 days, or until the rate of release of SF_6_ from the permeation tube is no longer stable. While repeatability of daily CH_4_ production is being improved as the methodology is refined (Deighton *et al.*, 2013), SF_6_ remains a very demanding method to get accurate emission measures over multiple days in individual animals.

Other systems that measure (or estimate) emissions over multiple short periods per day with minimal operator input have been developed. These include measuring all emissions from animals in short-term confinement; that is, Portable Accumulation Chambers (PAC; Goopy *et al.*, 2011), monitoring eructations in feeding stations (Negussie *et al.*, 2012) or voluntary milking systems for dairy cattle (Garnsworthy *et al.*, 2012; Lassen *et al.*, 2012), or Greenfeed monitors (GEM). A hand-held laser has been used to estimate CH_4_ flux indirectly from dairy cattle (Chagunda *et al.*, 2013). All of these methods, except PAC and GEM, measure concentrations, and assume that they have a constant recovery or little drift, and are therefore accurately reflecting gross flux from the animal over the recorded period. Similarly, all short-term estimates also assume that there is a high genetic correlation with longer term measurements and that this is essentially independent of when the animals are recorded. Average CH_4_ emissions in various units, heritability estimates, where known, and various repeatability estimates for example across days, across periods and across rounds are shown in Supplementary Table S1 for cattle and in Supplementary Table S2 for sheep. There are a wide array of variables including; system (RC, SF_6_, laser, GEM or PACs), diet (composition and particle size), feeding level (*ad libitum* or at a proportion of maintenance) and experimental period. Despite this, gross CH_4_ production and repeatability estimates are not so different. However, MY is variable with a noticeable difference between studies where animals are fed at a proportion of maintenance versus those that are fed *ad libitum*. Those fed at maintenance are theoretically estimating CH_4_ emission per live weight as much as CH_4_ emission per unit intake; CH_4_ emissions increases with live weight, and thus the ratio measure could be similar across time points in maintenance fed studies.

In summary, daily enteric emission is principally constrained by the quantity and fermentability of the feed consumed; but an understanding of within-day and between-day variances is required to ensure the emission data collected reflects the long-term CH_4_ phenotype of a ruminant. When collecting records for selective breeding, it will often be a choice between accuracy of the phenotype and number of records. In the case of gross CH_4_ production the most accurate method would be the RC method, but in order to generate enough data to do selective breeding and make recordings in practice, this method has limitations. Alternately, compared to RC, spot breath samples taken during milking in dairy cattle might be less accurate phenotypes for selective breeding, but can generate a large number of individual animal records. A genetic and environmental correlation structure between these methods together with 1 h RC methods, SF_6_ and other methods is needed and would allow merging of data to generate enough data for use in selective breeding.

## Implications for measurement

Three messages on repeatability emerge from Supplementary Table S1 and S2. The repeatability of daily CH_4_ emissions is highest between RC measures made on consecutive days, but diminishes as time between measures increases. Repeatability of CH_4_ emission is lower for short term measurement systems (e.g. PACs) relative to RC system. Consequently, more measures will be required from short-term measurement methods to capture variation within a day, but multiple samples across many days offers additional information about the robustness of the emissions phenotype that is not normally obtained by RC studies made only over 1 to 3 days. So far, we have not been able to source sufficient structured data from these methods and protocols to develop a common procedure for measurement of rate of CH_4_ emissions capable of being used for genetic selection.

McEwan *et al.* (2012) assessed the usefullness of multiple 1 h measures of emissions compared to 22 h RC measures using 684 sheep and found a high genetic correlation between 24 h emission measure and a 1 h emission measure (0.89 for g CH_4_/day and 0.76 for MY). They estimated there is little difference in estimates of CH_4_ emissions and MY by measuring animals twice in a RC, 14 days apart, or by measuring an animal four times for 1 h, 14 days apart. Such assessments indicate that using a range of measurement technologies is possible, but the intensity of sampling required and number of animals needing to be measured will be different for each system used.

It has been calculated that 3×1 h PAC measurements will be as useful at describing CH_4_ production rate as one RC measure for 1 day (Bickell *et al.*, 2011). Defining this comparability is a key requirement for developing measurement protocols of equivalent power to use in genetic selection.

Pinares-Patiño *et al.* (2011) showed that groups of animals selected to be high or low MY when consuming 2.2× maintenance lucerne pellets retained their ranking when fed lucerne and concentrate pellets. Subsequently they (C.S. Pinares-Patiño personal communication) demonstrated that with five different diets the groups remained different in MY, although individuals in the groups sometimes re-ranked (Table 1). Similar results were obtained by Michal *et al.* (2013) from growing beef heifers fed three different diets. This suggests that using a standard diet to assess rank of animals for MY is useful and the rankings are likely to hold across a range of production diets. The data also suggest that the differences in MY between animals in high and low MY groups (and therefore individuals) are greater when they are eating a more digestible diet. This suggests that the discriminatory power of a phenotype test could be expanded by feeding a mixed ration of forage and concentrate, although this requires testing with more animals.

**Table 1:** Consistency of response of sheep selected on basis of methane yield (g CH_4_/kgDMI) across time and a range of diets (C.S. Pinares-Patiño personal communication)

		CH_4_ yield (g/kg DMI)		
Time of measurement	Diet (fed at 1.3 to 1.6 M)	Low group (*n*=10)	High group (*n*=10)	% Difference between high and low group
August 2008	Grass silage	17.8	19.2	7.8
May 2009	Fresh grass	22.5	24.4	8.4
June 2009	60% Forage, 40% concentrate P	18.6	23.6	27.4
January 2010	Fresh grass	22.2	25.3	13.8
March 2010	40% Forage 60% concentrate P	8.9	12.8	43.8

## Breeding to reduce CH_4_ emissions from livestock

Genetic selection provides a reliable route towards permanent and cumulative reductions in quantitative traits such as enteric CH_4_ emissions.

To justify investment of effort and money in developing protocols for measurement of emissions to support genetic improvement in a CH_4_ trait, it is worth summarising evidence supportive of this breeding strategy (Lassey *et al.*, 1997). Genetic diversity in a range of digestive parameters likely to be associated with enteric CH_4_ production was apparent when reviewed in 2002 (Hegarty, 2004). The prospect for selection for a CH_4_ trait was initially investigated by multiple groups; some identified variation in CH_4_ traits amenable to animal selection (Robinson *et al.*, 2010) and some did not (Münger and Kreuzer, 2008). More recent research in 530 beef animals (Donoghue *et al.*, 2013) and 1225 sheep (Pinares-Patiño *et al.*, 2011 and 2013a) is increasingly supportive of CH_4_ traits being heritable with improvement by direct selection achievable.

Based on records of 1277 pedigreed sheep, estimated heritability and repeatability of CH_4_ across days, rounds and years, using the total 24 h measurement were 0.29±0.05 and 0.13±0.03 for gross CH_4_ production (g/day), and MY (g /kg DMI), respectively (Pinares-Patiño *et al.*, 2013a). There were high repeatabilities across consecutive days. Across rounds and across years the repeatability estimates were lower than for consecutive days, but, relatively stable. Estimation of genetic and phenotypic correlations with some of the main New Zealand production traits; weaning weight at 3 months, live weight at 8 months, fleece weight at 12 months (FW12), eye muscle depth and dag score (accumulation of faeces on the perineum region) at 3 or 8 months of age show that correlations with MY are low or close to zero, the only exception was FW12. The negative genetic and phenotypic correlations of FW12 with MY (−0.32±0.11 and −0.08±0.03, respectively) imply that selecting for increased hogget fleece weight would in part result in lower CH_4_ yield.

Results from Donoghue *et al.* (2013) on Australian Angus beef cattle showed very similar heritabilities. Based on 530 pedigreed cattle, fed at a proportion of maintenance (1.2×), heritability estimates for gross CH_4_ production (L/day), and MY (L/kg DMI) were 0.40±0.11 and 0.19±0.10, respectively. Genetic and phenotypic correlations of gross CH_4_ production with eye muscle area were 0.17±0.29 and −0.01±0.05, respectively. With MY, the genetic and phenotypic correlations were −0.02±0.30 and −0.03±0.05, respectively.

Both studies are based on 24 h RC measurement with known feed intake. However, the cost of routinely measuring CH_4_ emissions using RC is thought to be prohibitive for a testing programme using industry animals. Therefore, protocols for measuring or estimating CH_4_ production and feed intake are required that need less time and cost. It has to be kept in mind that phenotype recording of feed intake or DMI is most limiting in commercial condition and generally only recorded on experimental farms.

In the longer term, it may be possible to incorporate genomic information to estimate genomic breeding values (GEBVs) for CH_4_ emissions into breeding schemes (Meuwissen *et al.*, 2013). For GEBVs to be implemented, a reference population of several thousand genotyped industry relevant animals, with the CH_4_ phenotype measured, is required to provide initial estimates of the contribution of each genomic region to the expression of the phenotype under investigation (Calus *et al.*, 2013). Similarly, selection on GEBVs for correlated indicator traits can be used where it is impractical to directly measure CH_4_ on enough animals to establish a reference population. Finally, there must be an economic (and/or social) incentive to breed animals with the trait which is incorporated in the selection objective, so that the CH_4_ trait receives the appropriate weighting in any breeding programme.

There is already on-going improvement in emissions intensity that is CH_4_ emissions per unit product, arising from genetic selection for current production traits (Capper *et al.*, 2009; Wall *et al.*, 2010; Hayes *et al.*, 2013). One could therefore argue that further research investment into this area (i.e. selection for reduced intensity of CH_4_ emissions) is not necessary. However, selection solely on productivity traits such as live weight gain and/or milk production will increase feed intake and CH_4_ emissions per animal and hence total CH_4_ emissions unless a physical or economic constraint is imposed on total emissions. For dairy products, there is a market constraint on total production which has resulted in an increase in productivity per cow and a decrease in number of animals. This may suit some industries, but poses the question ‘is it possible to increase productivity and reduce CH_4_ emissions per animal at the same time?’ This could be achieved by reducing MY that is CH_4_ per unit feed consumed, and/or decreasing DMI provided that there is no concomitant reduction in productivity or increase in feed consumption. Selection on MY provides options to either reduce emissions while holding net enterprise feed consumption constant, or alternatively, allowing intake to increase supporting a production boost per animal without raising total emissions. Early results from a number of studies around the world, suggest that MY is both a heritable and repeatable trait (e.g. Pinares-Patiño *et al.*, 2013a). However, the means by which the host influences fermentation in the gut to affect CH_4_ production is still largely unknown. The extent to which genetic selection can be used to reduce MY is also not known. The methods by which CH_4_ emissions of individual animals can be measured are an important factor because the method used to measure the CH_4_ trait will also influence the resulting genetic parameters and is therefore an integral part of the selection programme. Besides, caution should be taken for ratio traits, as the genetic parameters may not truly represent the trait under consideration, because there is always extra variability of the denominator trait.

It is also important to remember that fertility and longevity have a huge aspect in the overall environmental impact of livestock, and therefore improved fertility and longevity through breeding and management will also be important mitigation strategies (Cottle *et al.*, 2011).

## Understanding animal variation in CH_4_ production over time

### Sources and transfer of CH_4_ within the ruminant

While CH_4_ is produced in both the reticulo-rumen and the hindgut, some transfer within the animal occurs before the CH_4_ is emitted. For example, in ewes eating lucerne, 97.5% of CH_4_ emission was voided via the oesophagus and lungs and only 2.5% via flatus, despite 23% of CH_4_ production occurring in the lower gut, presumably because of absorption of hindgut CH_4_ into the blood (Murray *et al.*, 1976). Cattle studies have shown the proportion of CH_4_ derived from the hindgut increases with feeding level (Hofmeyr *et al.*, 1984). Most of the CH_4_ leaving the rumen in oesophageal eructation is subsequently drawn into the lungs and then emitted in exhaled breath; although some rumen produced CH_4_ is also absorbed into the blood and diffuses into the lungs without passing up the oesophagus. This has been confirmed by dosing and radiotracer studies (Dougherty *et al.*, 1964; Heywood and Wood, 1985). The fraction of CH_4_ absorbed into the bloodstream from the gastrointestinal tract decreases as volume of eructated gas increases; also when an animal is not ruminating (Hoernicke *et al.*, 1965); and also after feeding (Hoernicke *et al.*, 1965). Studies with tracheotomised cattle have revealed that before feeding, 25% to 94% of the total CH_4_ emission (flatus not included) was by exhalation, whereas after feeding exhalation is reduced to 9% to 43% of emissions.

Cattle eructate every 40 to 90 s and take between 25 and 40 breaths per minute (Mortola and Lanthier, 2005), although the frequency of eructation peaks is reduced when drinking (Hegarty, 2013). As breathing frequency in cattle oscillates within a day and varies largely between animals (Piccione *et al.*, 2004), differences in gas excretion mechanisms (eructation, tracheal inhalation, exhalation and expiration) might differ considerably among individual animals.

**Figure 1:**
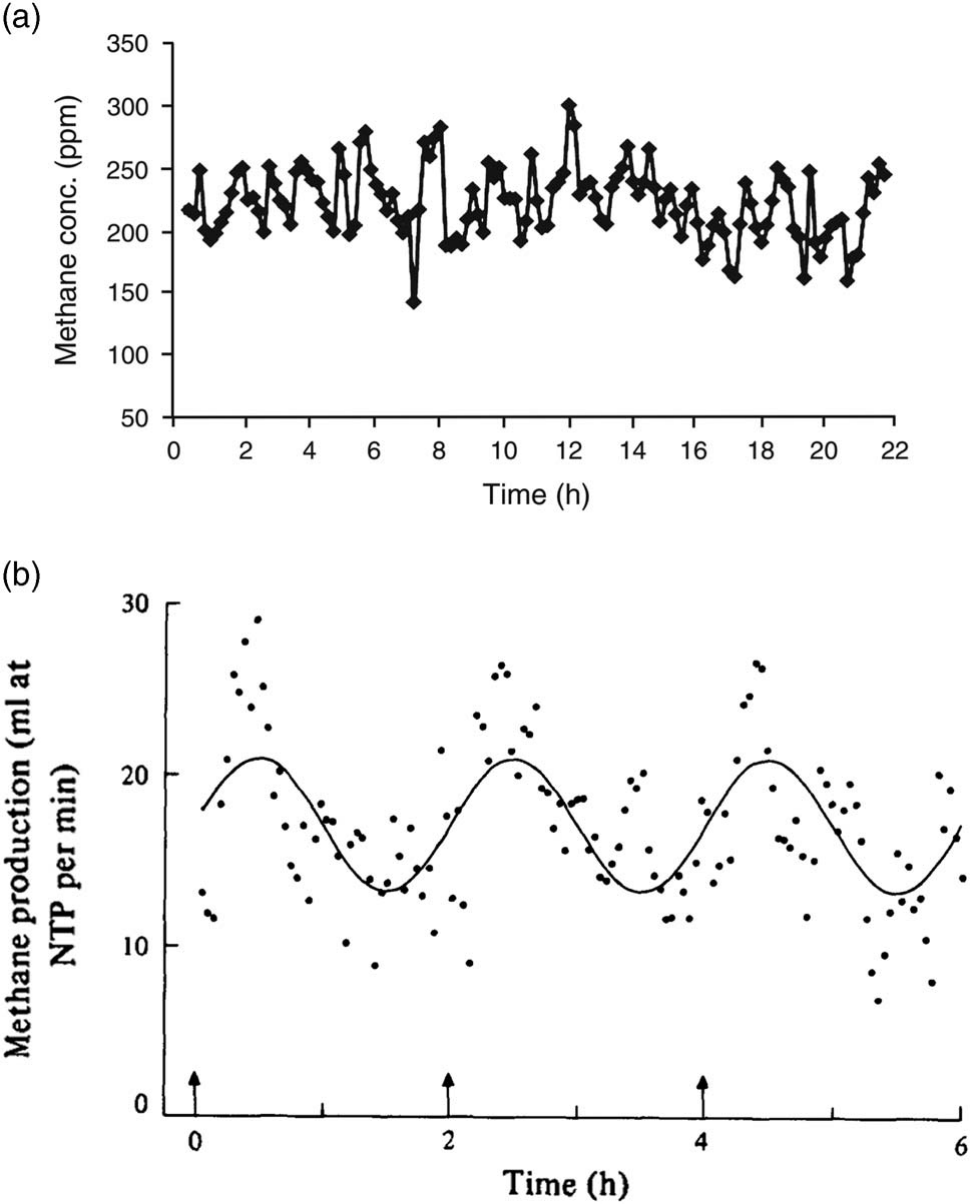
Time course of (a) methane concentrations (ppm) in respiration chambers (reproduced Nolan *et al.*, 2010, figure 1a), and (b) methane production (ml/min) (reproduced from Mathers and Walters, 1982, figure 2a), of sheep fed using an automated feeder at 2-h intervals.

While the proportion of CH_4_ entering the lungs by absorption or by inhalation varies, the important value is the absolute quantity and constancy of CH_4_ leaving the mouth and nose. Large oscillations in CH_4_ release rate (but not necessarily methanogenesis rate) are observed during CH_4_ measurements. Animal position and activity is known to affect pooling of gas in the rumen (McCauley and Dziuk, 1965), and pooling of gas in the rumen may be part of the reason that variable short term CH_4_ production rates are seen during RC studies even from animals fed at 2 h intervals (e.g. Figure 1a: Nolan *et al.*, 2010; Figure 1b: Mathers and Walters, 1982). Enteric CH_4_ production rate varies widely over 2 h intervals (Figure 1b), potentially contributing to a highly variable estimate of emission rate if measurements are short term. Mathers and Walters (1982) acknowledged ‘violent short-term variations were evident in the plots of the observations’, so emission rates were averaged, over various periods, to generate smoother emission profiles. Poor in-chamber mixing of air can cause similar variability in emission rates assessed over the short term (Gardiner and Coleman, 2013).

### Diurnal and longer term emission cycles

In the grazing environment, ruminants are considered to ingest most of their feed in morning and late-afternoon feeding sessions (see Gregorini, 2012 for recent review). Emulation of this pattern in RCs (Robinson, 2009) shows a biphasic diurnal CH_4_ emission pattern, consistent with timing of feed intake, but there was no difference in either total daily emission or MY when feed was provided in a single meal or as four equal meals in the morning and four equal meals in the afternoon. Murray *et al.* (2001) found a similar pattern of biphasic emissions in grazing sheep using a polytunnel.

A number of studies offer evidence of repeatability of emissions over prolonged periods, but the repeatability is confounded by the variations in pasture cover that occur with changes in season (Knight *et al.*, 2008; Münger and Kreuzer, 2008), so do not reflect innate repeatability of emission by the animal as would occur if the same diet was fed for a prolonged period.

Recent sheep genetics research provides evidence of repeatability over extended time intervals when a consistent diet is fed (Pinares-Patiño *et al.*, 2013a) and confounding with changes in feed composition do not occur.

## Indirect selection to reduce emissions

Measuring CH_4_ emissions directly from animals is difficult and thereby hinders direct selection on reduced CH_4_ emission. However, improvements can be made through selection on associated traits (e.g. RFI), volatile fatty acids (VFA), milk composition or through selection on CH_4_ predicted from feed intake and diet composition.

### VFAs

The rumen microbial population converts the host ingested food in the rumen into CO_2_, hydrogen (H_2_), VFA and microbial cells. The host absorbs the VFA across the rumen for its own use and rumen methanogens act on the H_2_ to produce CH_4_. High H_2_ concentrations are thought to stimulate methanogenesis while suppressing production of acetate and VFA in general, while low H_2_ concentrations will stimulate VFA production, especially acetogenesis but suppress methanogenesis. VFA are thus a potential proxy for estimating CH_4_ emissions. For sheep, Pinares-Patiño *et al.* (2013b) measured 1081 animals for VFA soon after exit from RCs. There were high genetic correlations (>0.78) of MY with log_e_ mM VFA concentrations. Genetic correlations are lower, but still moderate, when VFAs were expressed as molar %.

For cattle, Herd *et al.* (2013) measured VFAs and other parameters from 532 young Angus bulls and heifers soon after exit from the RCs (at least 12 h post feed consumption). Pearson correlation coefficients with CH_4_ production (L/day), MY (L/kg DMI) and CH_4_ intensity (L/kg live weight gain) were estimated. There were correlations of 0.40 with MY and CH_4_ emission intensity, but correlations with gross CH_4_ production were almost zero. Other studies (Robinson *et al.*, 2010), suggest that VFA concentration has limited utility in predicting CH_4_ emissions, although VFA production rate may be useful (McPhee and Hegarty, 2008). This contrasting evidence indicates considerable work is still required before the utility of VFA as an indicator of CH_4_ emissions can be realised.

### Prediction form mid-infrared spectra of milk samples

Mid-infrared spectra (MIR) of milk samples are generated routinely by national and commercial laboratories for prediction of milk composition during milk recording. Therefore, any approach that utilizes this information can immediately be implemented but also applied retrospectively to already analyzed samples with the spectral data stored. *In vivo* experiments performed using the SF_6_ method showed that it is possible to estimate CH_4_ emissions of lactating dairy cows from MIR spectra of milk samples (Dehareng *et al.*, 2012). A possible delay between a variation in CH_4_ emission and an onset in milk response was mentioned by these authors. These preliminary results suggest the possibility to predict individual CH_4_ emissions, allowing at least inventory type of assessments at a farm level or at a regional scale. With more collaboration and additional data, an improved equation could be generated. Predictions could then become robust enough to use MIR spectra to identify individually low-CH_4_-emitting cows and to develop selection and management tools to reduce CH_4_ emissions.

### Prediction from feed intake and diet composition

The objective of a Dutch study was to establish phenotypic and genetic variation in predicted CH_4_ output, and to determine the potential that genetic selection has in reducing CH_4_ emissions in dairy cattle (de Haas *et al.*, 2011). Records on daily feed intake, weekly live weights and weekly milk productions were available from 588 heifers. Along with RFI, predicted CH_4_ emissions (PME, g/day) and fat and protein-corrected milk production (FPCM, kg/day) were estimated. The estimated heritabilities for PME and RFI were 0.35 and 0.40, respectively. The positive phenotypic and genetic correlations between RFI and PME indicated that cows with lower RFI have lower PME as well (estimates ranging from 0.18 to 0.84 in different periods of the lactation). However, the association between these indicator traits and true CH_4_ output is unknown. It is still possible to decrease CH_4_ production of a cow by selecting more efficient (low RFI) cows, and the genetic variation suggests that reductions in the order of 11% to 26% in 10 years are theoretically possible, and in a genomic selection programme even higher. However, as stated previously, it is essential to ensure selection on production does not increase feed intake and CH_4_ emissions per animal and hence total CH_4_ emissions.

## CH_4_ in a genomic selection programme

CH_4_ emissions (as g CH_4_/day or MY) certainly fit the description of hard to measure traits. Methods currently available are expensive and time consuming (RCs and SF_6_) and subject animals to artificial environments. Those that measure animals in production situations (pasture, feedlot or dairy feeding station) sample CH_4_ for only a part of a day and require repeat measurements (PACs, Sniffers or GEM) and in some cases calculation back to known standard procedures. Those methods of estimating CH_4_ emissions that rely on computation of differences between feeding standards and production account for only part of the potential variation in CH_4_ emissions between animals.

Genomic selection opens the possibility to efficiently select for hard to measure traits. It is progressively being used to increase rate of genetic progress for production traits that are measured late in life (e.g. meat yield and quality), expensive to measure (e.g. RFI) and are sex linked (e.g. milk production and quality). In the dairy and increasingly in the beef and sheep industries leading sires are routinely genotyped and GEBVs are used in making selection decisions. It is doubtful that adding the cost of genotyping onto a population in which CH_4_ is measured would be cost effective, but by using industry animals which have measured production traits and have been genotyped it would be possible to estimate GEBVs for CH_4_ emissions. This is predicated on having a large reference population, where CH_4_ emission levels can be measured cheaply and genome wide DNA marker effects have been estimated, to establish the prediction equation for marker effects.

**Figure 2:**
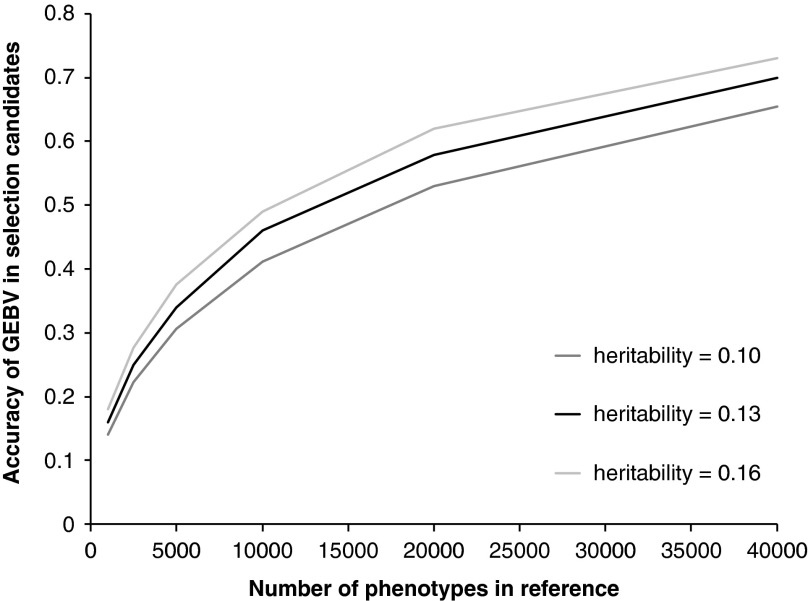
Accuracy of genomic estimated breeding values (GEBV) for methane yield (MY) in selection candidates as a function of heritability of the trait and number of animals with phenotypes in the reference population. Estimates of heritability of MY in sheep were obtained from Pinares-Patiño *et al.* (2013a).

The key question is how large does this reference population have to be, that is, how many animals need to be measured for CH_4_ and genotyped with the genome wide marker panels? Daetwyler *et al.* (2008), Goddard (2008) and Hayes *et al.* (2009) have all derived deterministic formula to estimate the accuracy of GEBV that could be achieved given the size of the reference population, the heritability of the trait and the effective population size. The accuracy of genomic selection for selection candidates (i.e. animals with a genotype, but no measured phenotype) with increasing size of reference population is shown in Figure 2. This was derived from the heritability of MY of 0.13 (Pinares-Patiño *et al.*, 2013a) and an effective population size of 150 using the procedure described by Hayes *et al.* (2009). This graph assumes perfect linkage disequilibrium between the single nucleotide polymorphisms (SNP) and quantitative trait loci, which is unlikely for the current available chips and thus the graph will asymptote to the proportion of variance explained. For example, for dairy cattle using the Bovine 50 K SNP chip this would be 90%. The estimates also assume unrelated individuals, if individuals were related, particularly the selection candidates and the reference population, the accuracy would be greater, as this is effectively reducing the effective population size. Finally, if the individuals in the reference population were progeny tested, this would make the ‘heritability’ of the trait much higher and thus would require fewer animals genotyped to achieve the same accuracy, however, the total number of animals measured for CH_4_ to achieve the same accuracy would stay the same.

Because MY is a new trait, it would be anticipated that even low initial accuracy will be useful to industry. As further animals are phenotyped the GEBVs would become increasingly useful. It remains to be determined if MY is independent of other (production) traits. If it is, then adding information from the GEBVs for MY into a selection index is relatively straightforward.

The number of animals with phenotypes in the reference population required to obtain GEBVs of high accuracy for MY is large and almost certainly exceed the resources available in any one country. To overcome these limitations an international effort is required to bring together data on production, feed intake and CH_4_ emissions of ruminants.

## Potential reduction in CH_4_

Although genetic selection is possible, the potential magnitude of selection for MY is unknown. Pinares-Patiño *et al.* (2013a) report a difference of 8% in MY between sheep after one generation of selection for and against MY. The extent to which variation in MY can be exploited, depends on the stability of the underpinning relationships with production traits. The best way to incorporate this is with a selection index that includes traits related to production, functional traits and environmental impact. This will result in a slower response to selection for all traits, but in a good overall response to the overall breeding goal. The mechanisms that contribute to genetic variation in MY of individual animals may include: reduced fermentation of organic matter in the rumen (due to shorter retention time of digesta; Pinares-Patiño *et al.*, 2011 and smaller rumen volume; Goopy *et al.*, 2014), instability of fermentation (natural occurring defaunation; Faichney and Graham, 1996), different microbial populations in the rumen and potentially reductive acetogenesis (inferred from Faichney and Graham, 1996). The extent to which these combine to produce natural variation in MY is unknown, but data from measurement of MY in sheep using RCs suggest that the coefficient of variation is 10.3% (Pinares-Patiño *et al.*, 2013a) and for cattle 14% (Donoghue *et al.*, 2013). It would not be unreasonable to anticipate a response to long term selection to exceed 2 standard deviations from the mean, suggesting that a reduction of up to 25% in MY may be feasible through selection of livestock for low MY. Combined with potential reduction in CH_4_ emissions due to selection for low RFI, this suggests that a reduction in CH_4_ emissions of 40% to 45% may be possible through selection of individual animals on components that directly affect CH_4_ production. Differences in feed intake of 1.17 kg/day between beef cattle selected for and against RFI were observed after 2.4 generations, equivalent to a difference of 18 g CH_4_/day around a mean 180 g CH_4_/day or a 10% difference (Hegarty *et al.*, 2007). It remains to be seen if this is independent of productive traits, although in practice selection for reduced feed intake and CH_4_ emissions will be conducted using an index that includes production traits.

## Expectations of methods for measuring CH_4_

The key requirements of a methodology for measurement of CH_4_ production and MY of individual animals for genetic selection are, first, the methodology must provide a reliable measure of the true CH_4_ emission by the individual for the period of measurement and suitable for the production system under target. This requires that the recovery of CH_4_ emissions by the measurement procedure be consistent and preferably 100%. The RC, PACs, GEMs and SF_6_ all potentially meet these criteria (Table 2). Methods where recovery is <100% might be useful if they show consistent recovery and capture diurnal variance in emissions rate. These include GEMs and sniffers which permit losses of CH_4_ between animal and sensor.

**Table 2:** Summary of the main methodologies for individual methane measurements

Method	Robust	Intrusive	Cost	Throughput
Respiration chamber	Yes	Yes	High	Low
Short-term accumulation chamber	Yes	Yes, but easily managed with grazing animals	Low	High
Greenfeed monitors	?	Moderately, requires modified grazing pattern	High	Moderate
SF_6_	?	Yes for sampling, less so for grazing	High	Moderate

Second, the period of measurement (of CH_4_ and for MY, feed intake) and number of measurement periods should be sufficient to reliably rank sires for estimation of breeding values. In practice, this means multiple measures per animal. The optimal period and number of measurements will be determined by the pedigree structure of the data and the purpose of research. The repeatability of CH_4_ measurements in PACs is only slightly less than in RCs (Table 2; Pinares-Patiño *et al.*, 2013a). There is limited data to reliably estimate repeatability of CH_4_ emissions using SF_6_ and GEMs (Table 1), but it is anticipated that it would be less than in RCs. Having more progeny per sire will increase the accuracy of the estimate of sire EBVs and having more sires will improve the accuracy of the initial estimates of heritability. Finally, the measurement must be robust over time, as low cost as possible, not unduly influence animal behaviour and permit a high rate of data capture with low labour requirements. Ideally it should replicate the normal production system as far as possible.

## Conclusions

There is potential for adopting genetic selection and in the future genomic selection, for reduced CH_4_ emissions in ruminants. From this review it has been observed that direct measurement of CH_4_ emissions from RC, SF_6_ or PAC has proven underlying animal genetic variability. Subsequently, indirect indicators were explored through genetic correlations with CH_4_ trait. It can be concluded that indirect and genomic selection might be possible options for near future selection. CH_4_ emissions are a heritable and repeatable trait. CH_4_ emissions are strongly related to feed intake both in the short term (minutes to several hours) and over the medium term (days). When measured over the medium term, MY is a heritable and repeatable trait albeit with less genetic variation than for total CH_4_ emission (g/day). CH_4_ emissions of individual animals are moderately repeatable across diets, and across feeding levels, when measured in RCs. Repeatability is less when short-term measurements are used, possibly due to variation in time and amount of ingested feed before the measurement. However, repeated measurements add value; it is preferable the measures be separated by at least 3 to 14 days. This needs to be investigated further. Given the above issue can be resolved, short-term (over minutes to hours) measurements of CH_4_ emissions show promise. Finally, we believe that for short-term measurements to be useful for genetic evaluation, a number (between 3 and 20) of measurements will be required over an extended period of time (weeks to months).

There are opportunities for using short-term measurements in standardised feeding situations such as breath ‘sniffers’ attached to milking parlours or total mixed ration feeding bins, to measure CH_4_. We anticipate these are also subject to the caveats above about the use of short-term measurements. The measurement ‘protocol’ (i.e. how the animal and its feeding behaviour are managed before measurement) is more important than the technology used to make the CH_4_ measurement. While there is evidence that correlated and predictor traits exist for CH_4_ emissions the current level of knowledge is insufficient to recommend their use in genetic selection to reduce CH_4_ emissions. Genomic selection has the potential to reduce CH_4_ emissions and MY, however, measurements on thousands of individuals will be required. This includes the need to combined resources across countries in an international effort, emphasising the need for acknowledging the impact of the animal and production system on measurement of the CH_4_ trait during design of experiments. The ‘size of the prize’ when combining lower MY with selection for low RFI may result in a reduction in CH_4_ emissions of 40% to 45% and may be possible through selection of individual animals on components that directly affect CH_4_ production.

In summary we consider genetic and genomic selection offers a significant opportunity to reduce CH_4_ emissions from ruminants. However attention needs to be directed to a number of issues if short-term low-cost measurements are to be implemented in industry.

## Supplementary material

To view supplementary material for this article, please visit http://dx.doi.org/10.1017/S1751731115000968
